# The American Year Book of Medicine and Surgery

**Published:** 1900-07

**Authors:** 


THE AMERICAN YEAR BOOK OF MEDICINE AND SURGERY.—Being a yearly digest of scientific progress and authoritative opinion in all branches of medicine and surgery, drawn from journals, monographs and text-books of the leading American and foreign authors and investigators. Collected and arranged with critical editorial comments by Samuel W. Abbott, M. D.; Archibald Church, M. D.; L. W. During, M. D.; Alfred Hand, Jr., M. D.; M. R. Hatzell, M. D.; R. W. Wilcox, M. D.; Wyat Johnson, M. D.; Walter Jones, M. D.; David Reisman, M. D.; Louis Starr, M. D.; Alfred Strengel, M. D.; A. A. Stevens, M. D.; G. N. Stewart, M. D. Under the general editorial charge of George M. Gould, M. D., Medicine. Pages 656. Price, $3 each, net. Philadelphia: W. B. Saunders, 925 Walnut Street. 1900.

The plan of the Year Book since last year has been changed from a single volume to two volumes of less size. The change seemed, to the management, to be demanded on account of the large size and weight of the single volume.

The editor, Dr. George M. Gould, and his able corps of assistants, are making a most excellent annual. The popularity of the Year Book attests this.

